# A reinforcement learning approach for protein–ligand binding pose prediction

**DOI:** 10.1186/s12859-022-04912-7

**Published:** 2022-09-08

**Authors:** Chenran Wang, Yang Chen, Yuan Zhang, Keqiao Li, Menghan Lin, Feng Pan, Wei Wu, Jinfeng Zhang

**Affiliations:** Department of Statistics, Florida State University, Tallahassee, FL 32306-4330 USA

**Keywords:** Protein ligand docking, Reinforcement learning, A3C, Asynchronous advantage actor-critic model, Protein ligand binding mode prediction, Protein ligand binding

## Abstract

Protein ligand docking is an indispensable tool for computational prediction of protein functions and screening drug candidates. Despite significant progress over the past two decades, it is still a challenging problem, characterized by the still limited understanding of the energetics between proteins and ligands, and the vast conformational space that has to be searched to find a satisfactory solution. In this project, we developed a novel reinforcement learning (RL) approach, the asynchronous advantage actor-critic model (A3C), to address the protein ligand docking problem. The overall framework consists of two models. During the search process, the agent takes an action selected by the actor model based on the current location. The critic model then evaluates this action and predict the distance between the current location and true binding site. Experimental results showed that in both single- and multi-atom cases, our model improves binding site prediction substantially compared to a naïve model. For the single-atom ligand, copper ion (Cu^2+^), the model predicted binding sites have a median root-mean-square-deviation (RMSD) of 2.39 Å to the true binding sites when starting from random starting locations. For the multi-atom ligand, sulfate ion (SO_4_^2−^), the predicted binding sites have a median RMSD of 3.82 Å to the true binding sites. The ligand-specific models built in this study can be used in solvent mapping studies and the RL framework can be readily scaled up to larger and more diverse sets of ligands.

## Introduction

Protein–ligand docking is a molecular modeling technique that predicts the binding and binding affinity between a target protein and a ligand [1, 2]. As proteins function by interacting with other molecules, small molecule ligands are often used to bind to the active sites (or binding sites) of target proteins to modulate their functions [3]. In drug discoveries, protein–ligand docking is an important early step to finding potential drug candidates through structured-based drug design (SBDD). Given a target protein, potential binding ligands are searched in a ligand database to identify lead compounds, which may be further refined using other criteria. This computational approach can potentially help pharmaceutical companies find promising lead compounds much faster at a very low cost [4].

The protein–ligand docking (PLD) procedure can be described as a combination of searching algorithm and scoring function. The searching algorithm searches the space of the binding conformations between the ligand and the protein during the docking procedure, and the scoring function evaluates the goodness of a given binding conformation. Many methods have been developed in the past with various searching algorithms and scoring methods to address the docking problem. In general, there are three types of docking: rigid docking, flexible-rigid docking, and flexible docking [5]. Firstly, rigid docking assumes the receptor and ligand are rigid bodies, which implies their conformations would not change during the docking process. DOCK [6] and MS-DOCK [7] are rigid docking methods developed by shape matching. They search for the ligand pose and binding site according to the criterion that the molecular surfaces of the ligand and the binding site on the protein should match each other. ZDOCK [8] proposed a new scoring function that combines pairwise shape complementarity with desolvation and electrostatics to optimize the rigid docking process. Second, flexible-rigid docking considers the ligand’s flexibility while the protein’s conformation is fixed. Autodock [9] is a program that applies simulated annealing for the bound conformations of the ligand and a rapid grid-based method for the scoring. Other tools such as Autodock Vina [10] used iterated local search global optimizer to speed up the docking procedure and improve the binding mode predictions. Third, flexible docking assumes both receptor and ligand can change conformations during the docking process. GOLD [11] is based on the genetic algorithm and Goldscore function. Autodock Vina [10] also has the option that allows the flexibility of proteins.

Over the past decade, machine learning methods have been successfully applied to PLD and achieved state-of-the-art performances. Specifically, RF-Score [12] used the random forest to ensemble different structure-, knowledge-, and empirical-based features, and made a substantial improvement on scoring functions. Koppisetty et al. [13] built support vector machine-based scoring functions by utilizing protein–ligand interaction or ligand-based descriptors. Moreover, task-specific models were trained with better performances using an ensemble machine learning method and a gradient boosting decision tree-based docking method [14]. Multi-task learning using a deep neural network (MT-Net) also showed superior performance than conventional scoring functions [14]. Wang et al. developed feature functional theory—binding predictor (FFT-BP) [15] where a large number of features were first extracted using physical models and then binding affinity models were trained using machine learning methods including deep learning. In DeepVS [16], a deep learning method was developed to learn atom embeddings without feature engineering, and achieved state-of-the-art performance on a benchmark decoy dataset. Finally, solving the 50-year-old protein structure prediction problem by AlphaFold (Senior et al. [17, 18] in 2020 clearly demonstrated the potential of deep learning methods in computational structural biology. Unlike conventional machine learning techniques that require feature engineering, a great advantage of deep learning-based methods is that they allow raw data to be directly fed into appropriate networks to train powerful predictive models.

This manuscript proposes a new approach to address the protein–ligand docking problem by applying deep reinforcement learning (RL). RL is a type of machine learning method which deals with sequential decision-making. It is straightforward to combine the RL method with deep learning, and the resulting method is known as deep RL (DRL). The key elements in RL are the agent, environment, and reward where the agent takes actions in the environment to make changes to the environment, which provides feedback in the form of a reward based on the action. The *actor-critic (AC)* [19] is an RL algorithm that combines the policy-based and value-based RL methods. In the AC algorithm, the actor and critic models are trained simultaneously, making it possible to learn a binding site prediction model and a scoring function in one shot. The *advantage actor-critic (A2C)* [20] is an updated version of AC with advantage functions, and the *asynchronous advantage actor-critic (A3C)* [21] is an advanced version of A2C designed for parallel training. Since the actor model and critic model can satisfy the searching algorithm and scoring function requirements, it is feasible to use A3C as the framework with a deep neural network to address the protein–ligand docking problem.

Designing an effective RL framework for protein–ligand docking that addresses both the sampling algorithm and scoring function is more challenging than applying deep learning methods separately to the two problems. To the best of our knowledge, there has been very few RL-based deep learning model [22] on protein–ligand binding pose prediction. Current literature (Ye el al. [23] on ion positioning prediction focuses on predicting which residue that ion should bind to. We will only consider rigid docking in this project by placing the ligand at a random place far away from the protein at the beginning. The RL framework contains two models: 1) an actor model, which is a ProDCoNN (Fig. 4 structured deep neural network, will be trained to choose an action that brings the ligand to newly predicted binding positions; 2 a critic model with similar architecture to the actor model, which can produce an evaluation score, will be trained to evaluate the fitness of the predicted binding positions. In this study, the feasibility of applying RL on the protein–ligand docking problem will be explored. We focus on developing models for single atoms and small multi-atom ligands in this study and the models are ligand specific. The ligand-specific models can be used in solvent mapping studies [24] to assist structure-based drug design.

The rest part of this paper is organized as follows. Section 2 describes the details about the experimental data, including the source of the data and the preprocessing procedure. We also introduce the A3C algorithms for the protein–ligand docking problem and describe the training and testing procedures. In Sect. 3, we evaluate the performance of the proposed method on real protein–ligand binding data. In particular, we will focus on the effect of hyper-parameter settings, model architectures, and sample sizes to the model performance during the training and test processes. We conclude the paper with a discussion and summarization in Sect. 4.

## Materials and methods

**Fig. 1:**
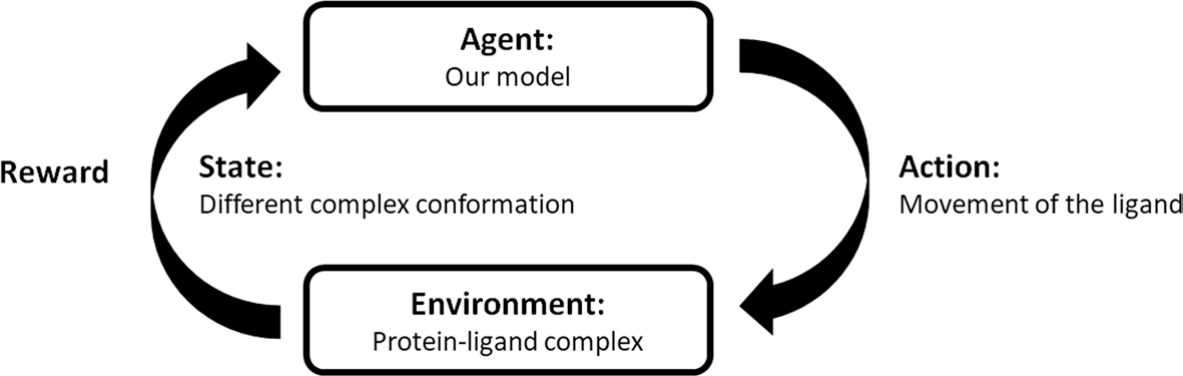
The learning loop of RL

Figure 1 shows an illustrative learning loop of RL in the context of the protein–ligand docking problem. Through the interaction between our model (the agent) and the protein–ligand complex environment, the model generates a movement on the ligand (the action), which leads to a different complex conformation (the state). Then, the environment outputs the new state as well as an immediate reward based on the action. In the next time step, this new state and reward are fed back to the agent to generate the next action on the state.

In this study, we generated several three-dimensional (3D) cubic gridded boxes to capture the local protein structure. Each box contains the whole true ligand position and the protein environment surrounding it. During the training process, the ligand of interest is placed at a randomly selected location in the box, and then it will move based on the guide from the model. Therefore, it is analogous to the maze problem in reinforcement learning: we need a model which is reasonable enough to guide the object in the environment to the goal and adjust the ligand pose to bind with the protein properly.

### Data format and preprocessing

We select protein–ligand complexes with some specific ligands (Cu^2+^, SO_4_^2−^) from the Protein Data Bank (PDB) [25, 26] with the protein sequence’s identity lower than 30%. Removing homologous sequences is commonly done in computational structure biology studies. Protein with similar sequences usually have similar backbone structures. Although we did not take the whole protein structures as input, certain biases may still be introduced if we include homologous sequences. The datasets analyzed during the current study are available in the RSCB the Protein Data Bank, https://www.rcsb.org/. All these structures are determined by X-ray crystallography with a resolution better than 2.0 Å and do not have any DNA/RNA/UNK molecules.

**Fig. 2:**
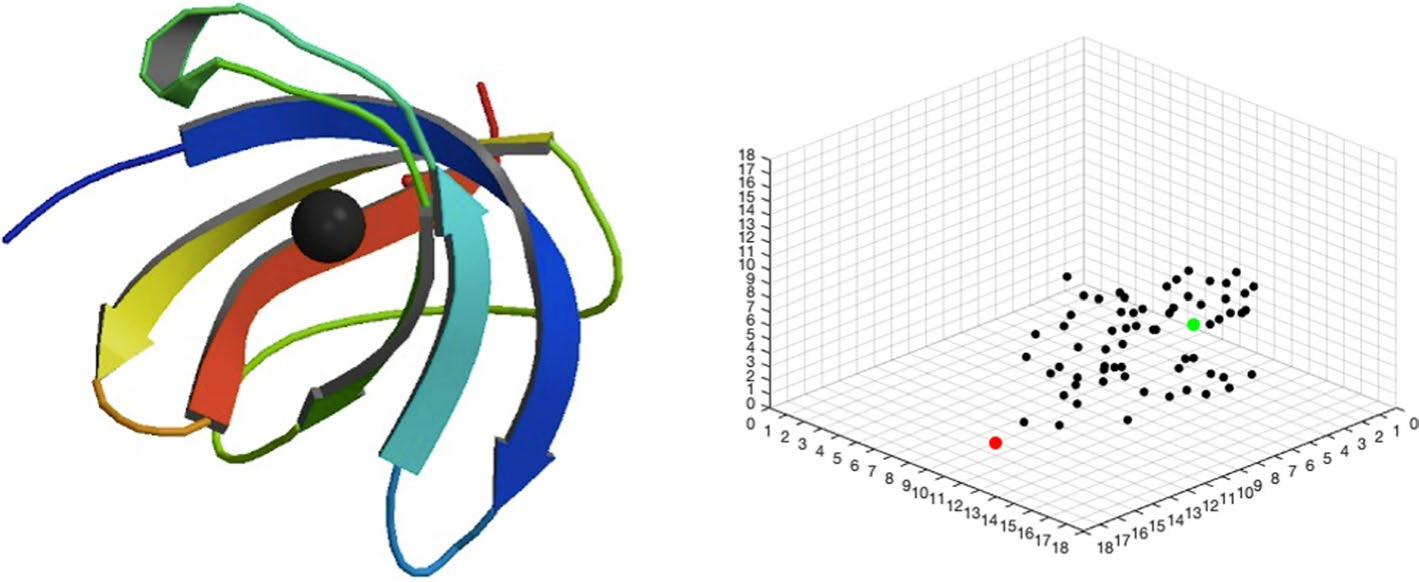
Left: The 3D structure of protein 2vb2. Right: The gridded box generated from protein 2vb2. The black points are atoms in the protein environment. The green point is the true ligand position where the ligand type is Copper ion. The red point is the starting ligand site

The cubic gridded boxes are generated from the protein–ligand complex data. Given the protein structure and the ligand on the true ligand position (the position of ligand as in the crystal structure from the PDB), we at first randomly rotate the whole structure, and then generate a point as the center of the 18 Å × 18 Å × 18 Å cubic box. This box is gridded with 1 Å unit size voxel; most voxels contain no more than one heavy atom. The whole ligand and part of the protein structure should be contained in the box. Finally, we randomly rotate the ligand and move it to a random place in the box as the starting ligand site. According to this data generation process, each protein–ligand complex can generate multiple cubic gridded boxes. The right plot of Fig. 2 shows a box taken from the protein 2vb2 [25, 27]. The true ligand position is the green point; that is, the Copper ion (Cu^2+^) ligand is there. The black points are atoms in the protein environment. The red point is the starting ligand site, which is randomly selected.

In this paper, different types of atoms will be put in different channels, so the dimension of the input data is $18 \times 18 \times 18 \times N$ where $N$ is the number of channels. In general, the total number of channels is $N = 21 + M$ where there are 21 types of atoms in the protein environment, and the additional $M$ types are from the ligand of interest. Gaussian smoothing will be applied to the atom to preserve atom radius information. We use a Gaussian smoother to spread the effect over 26 ($3 \times 3 \times 3 - 1)$ voxels around the voxel containing the target atom. Assuming the coordinate of the target atom is $\left( {x_{0} ,y_{0} ,z_{0} } \right)$, the Gaussian smoothing value at $\left( {x^{\prime } ,y^{\prime } ,z^{\prime } } \right)$ is:

$$\frac{1}{{\left( {2\pi r^{2} } \right)^{\frac{3}{2}} }} \times \exp \left\{ { - \frac{{\left( {x^{\prime } - x_{0} } \right)^{2} + \left( {y^{\prime } - y_{0} } \right)^{2} + \left( {z^{\prime } - z_{0} } \right)^{2} }}{{2r^{2} }}} \right\}$$

where $r$ is the Van der Waals radius of the atom in each voxel. We normalize these 27 voxels to get the final Gaussian smoothing values for all voxels influenced by the atom. Finally, we add up the smoothing values for all atoms in the same channel and obtain the $18\times 18\times 18\times N$ input data.

### Methodologies

Our reinforcement-learning-based protein–ligand docking method is constructed by using the *Asynchronous Advantage Actor-Critic* (A3C) algorithm [21]. We propose this new method to unify the ligand pose adjustment and score estimation under one framework. A3C is an actor-critic-based algorithm. The actor and critic models are designed as two ProDCoNNs (see Fig. 4) and are trained in the learning process. The actor model takes the current state as input and outputs an action, which can maximize the long-term reward on each state. The critic model also uses the current state as input, and produces a score based on the current actor model to measure the goodness of the chosen action. Ideally, the critic model is trained to predict the real long-term reward on each state. The key point for the A3C algorithm is a multi-threaded asynchronous model learner. Multiple sub-learners can train models independently and then send the gradients with respect to parameters back to the major environment to update them.

Thousands of chemical substances can form complexes with proteins and serve biological purposes. A ligand can be formed by either one or multiple atoms. This manuscript will show the natural protein–ligand complex experimental settings and results with ligand Copper ion (${\mathrm{Cu}}^{2+}$) and ligand Sulfate (SO_4_^2−^), respectively. We here simplify the problem by ignoring the atom collisions and bonds between atoms. The data setups and model structures will be discussed in the following sections.

#### Algorithms

**Fig. 3:**
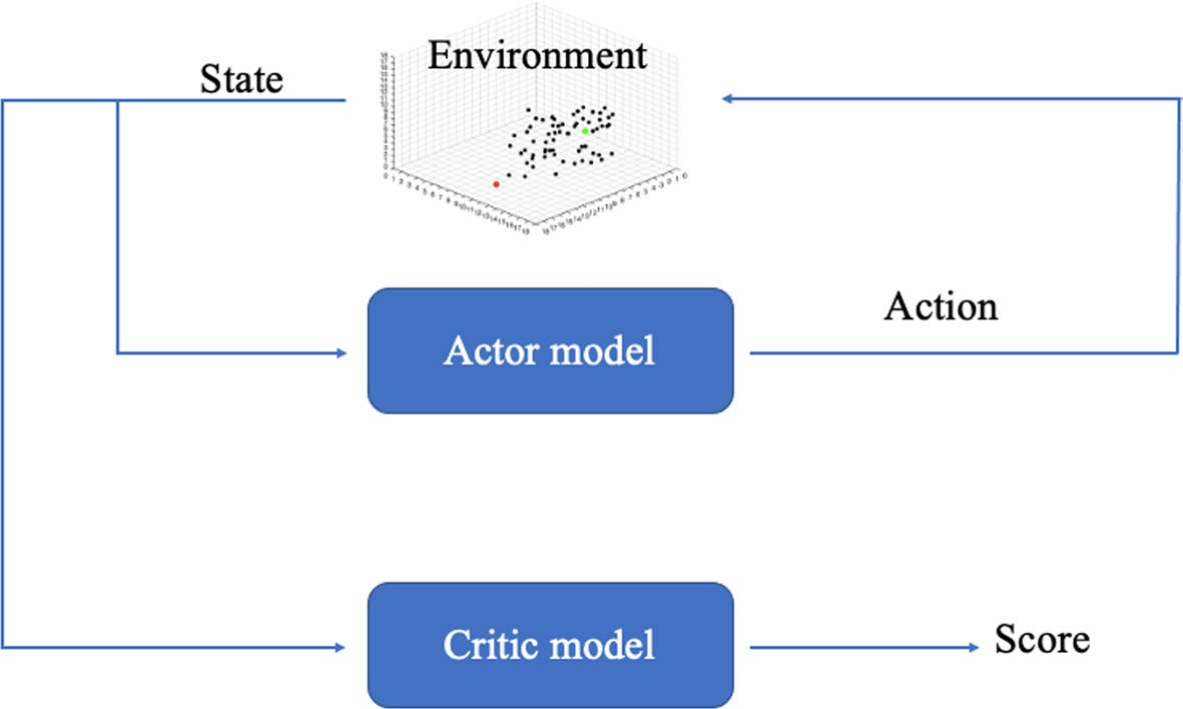
The flow of information in A3C

In this project, we combine the RL idea and the supervised learning idea. Based on A3C, in the beginning, we add one loop to feed a new cubic box so that the algorithm can train the model with a new environment in each episode. This method can help the actor and critic models learn general information about the protein environment and the docking process. A simple schematic for the flow of information is presented in Fig. 3. Through the interaction between the ligand and environment, the actor outputs action, and the critic model generates a score to evaluate the performance. Table 1 lists notation used in the framework. Algorithm 1 shows the detailed training process based on A3C. Some crucial elements in this algorithm are:

**Table 1:** Important notations in RL

Notations	Meaning
$s \in {\mathcal{S}}$	State
$a \in {\mathcal{A}}$	Actions
$r \in {\mathcal{R}}$	Immediate reward
$\gamma$	Discount factor
$G_{t}$	The long-term reward: $G_{t} = \mathop \sum \limits_{k = 0}^{\infty } \gamma^{k} R_{t + k + 1}$
$\pi_{\theta } (a|s)$	Actor model with parameters $\theta ;$ it is a distribution of action given the state
$V_{\omega }^{\pi } \left( s \right)$	Critic model with parameters $\omega ;$ it depends on the policy model and can output score

- Box: Cubic box contains protein atoms environment and a ligand on the starting ligand site.
- Action $A_{t}$: There are six actions for the ligand – moving forward and backward on each of X, Y, and Z axes. There are also six rotation directions, clockwise and counterclockwise, on each of X, Y, and Z axes. The ligand can choose a moving direction and a rotation direction in each step.
- Immediate reward $R_{t}$: The reward for step $t$ with an action leading the ligand to the step $t + 1$ is defined as:
  $$e^{{ - \frac{{{\text{RMSD}}\left( {s_{0} ,s_{t + 1} } \right)}}{18}}} - e^{{ - \frac{{{\text{RMSD}}\left( {s_{0} ,s_{t} } \right)}}{18}}} ,$$
  where RMSD is root-mean-square deviation, an average type of distance between atoms, $s_{0}$ is the coordinate of the true ligand position. If $R_{t} < 0$, $R_{t} \leftarrow R_{t} \times 2$ adds a penalty to force the ligand to walk in a better direction to the true position.
- $T_{MAX}$: The maximum number of steps that the ligand can move in the cubic box.
- $t_{max}$: The maximum number of steps that the algorithm needs to collect data to update the gradients with respect to parameters each time.

During the training process, given the ligand on the starting ligand site and the fixed protein atoms environment, we can generate a cubic box and feed it into the actor and critic models. Each time the ligand moves, we use the fixed protein atoms environment and the ligand on the current state to generate a new box, and then feed it into the models.
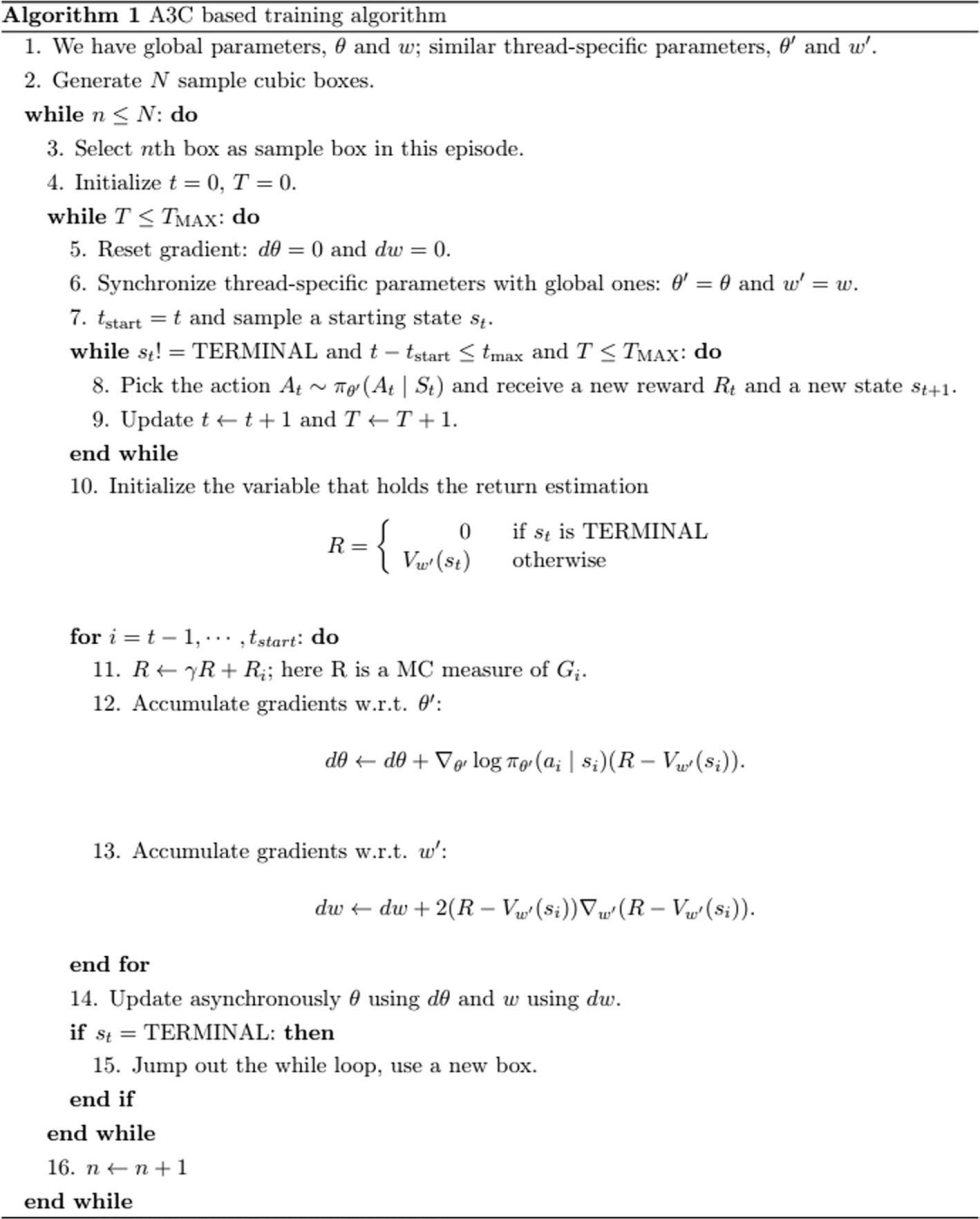


Unlike the traditional RL without any label information, Algorithm 1 is supervised. In the training stage, the immediate reward is defined based on the known true ligand position, which is unavailable in the test stage or in practical use. Algorithm 2 shows the details of the test process. Given the trained actor model and critic model, the ligand in the box can move according to the actor model predictions. The critic model will be used to stop the searching process in the box. If the step number is larger than the minimum steps, and the range of last $\delta$ critic outputs is less than the threshold value, then the actor will stop searching. In addition, if the step number reaches the maximum or ligand moves out of the box, then the test for the current box stops.
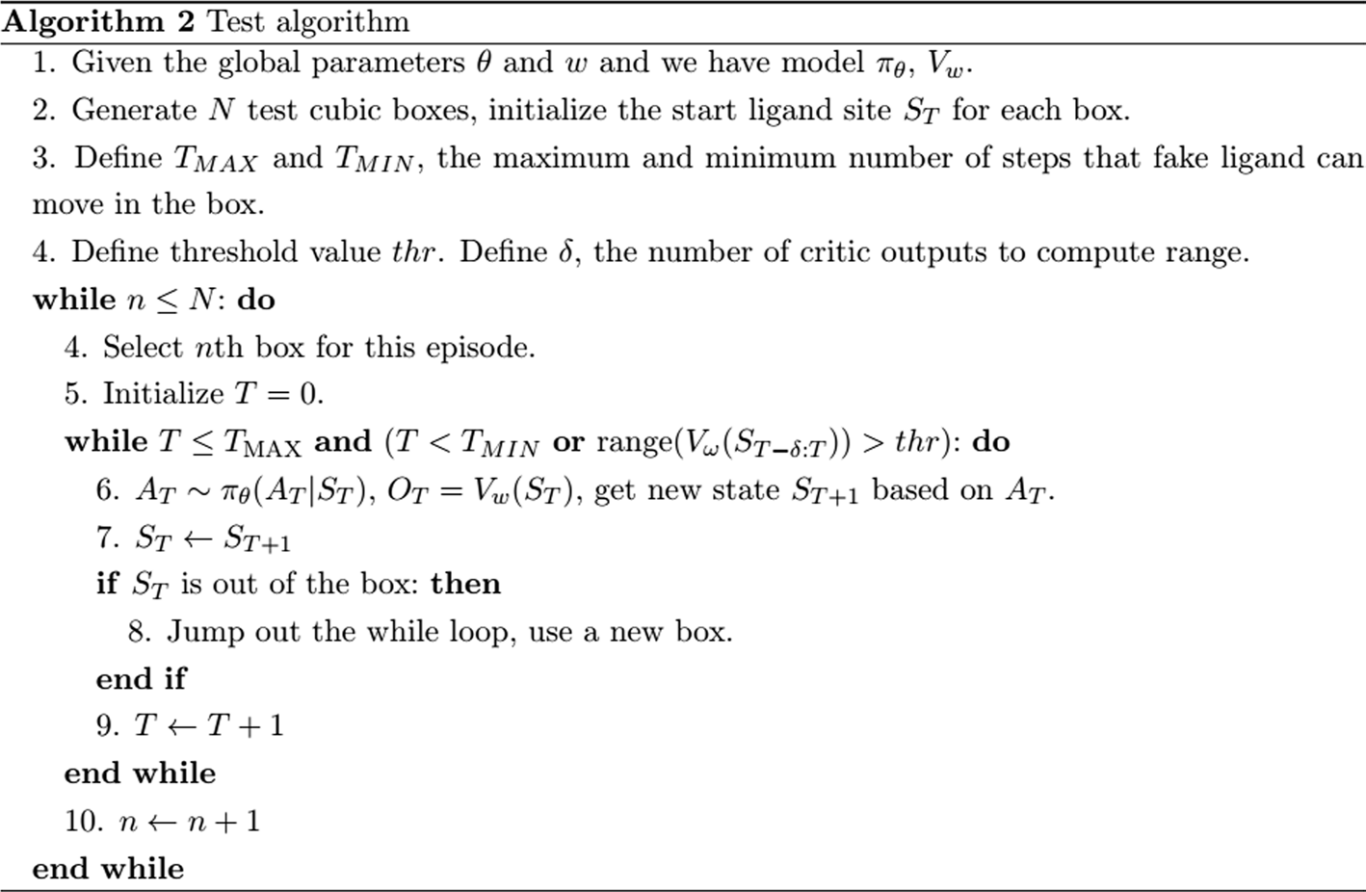


#### Copper ligand experimental settings

The data for protein–ligand complexes with the Copper ligand (Cu-ligand dataset) are selected from PDB. The protein sequence identity is lower than 30%. The cubic gridded boxes are generated based on the description in Sect. 2.1, and the number of proteins and boxes in the training and test dataset are presented in Table 2. In the training process, the learning rate for the actor model is 0.00005, and 0.0000001for the critic model. In each episode ${T}_{MAX}$, the maximum number of steps that the ligand can move is 600. The parameters of two models will be updated every ${t}_{max}=10$ steps with discount rate $\gamma$ as 1. In each step, the ligand can move 0.1 Å. In the test process, ${T}_{MAX}$ is set as 600, which is the same as that in the training process. The ligand moves 0.1 Å for each step, and it must move at least ${T}_{MIN}$ = 300 steps unless it moves out of the box. Also, we let threshold be 0.3 and $\delta$ equal 50, which indicates that we utilize a 50-step window to check the convergence of the critic distance.

**Table 2:** Proteins and boxes number of Cu-ligand dataset

	Training dataset		Test dataset	
	No. of proteins	No. of boxes	No. of proteins	No. of boxes
Cu-ligand dataset	50	20,000	8	4000

**Fig. 4:**
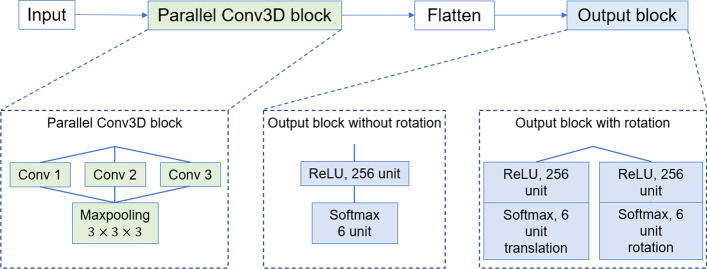
The architecture of actor model for the Cu-ligand dataset and SO_4_^2−^-ligand dataset. The Cu-ligand dataset uses output block without rotation, and the SO_4_^2−^-ligand dataset uses output block with rotation

We refer to the parallel Convolutional Neural Network (CNN) structure from ProDCoNN [1, 2] for actor and critic models, shown in Fig. 4 for the Cu-ligand dataset. The 6-layer model structure for the actor model is given below:

1. Input layer: Dimension is $18\times 18\times 18\times 22$, with 21 types of atoms in the protein environment and Copper ligand in the 22nd channel.
2. A parallel 3D convolutional layer which consists of three independent 3D convolutional layers: 4 filters with size $2\times 2\times 2$, 8 filters with size $3\times 3\times 3$, 8 filters with size $4\times 4\times 4$. Their border modes are the same, which can generate the feature maps with the same dimension as input.
3. Max pooling layer: $3\times 3\times 3$.
4. Flatten layer.
5. Dense layer with unit number 256 and ReLU activation.
6. Output layer with unit number 6 and Softmax activation.

The ${\mathrm{Cu}}^{2+}$ ligand is just a single atom. Therefore, the ligand rotation will not be considered, and the output layer should just result in the direction probabilities. The architecture of the critic model is the same as the actor model except for the output layer. In the critic model, the output layer has only one unit, and the activation is “tanh” to generate a value for estimating the true long-term reward.

#### Sulfate ligand experimental settings

Same as the Cu-ligand dataset, the Sulfate ligand dataset (SO_4_^2−^-ligand dataset) is also selected from PDB with protein sequence identity lower than 30%. The numbers of proteins and boxes of the training dataset and test dataset are presented in Table 3. In this multi-atom ligand training process, the learning rate for the actor model is 0.000005, and for the critic model is 0.0000001. In each step, the ligand can move 0.1 Å and rotate 1°. In addition, all other hyper-parameters in both training and test processes, such as ${T}_{MAX}$, ${t}_{max}$, $\gamma$ and thread number, are the same as those in the Cu-ligand dataset experiment.

**Table 3:** Proteins and boxes number of SO_4_^2−^-ligand dataset

	Training dataset		Test dataset	
	No. of proteins	No. of boxes	No. of proteins	No. of boxes
SO_4_^2−^-ligand dataset	166	24,900	23	4600

One special feature in the SO_4_^2−^-ligand dataset experiment is the method to compute the distance between two ligands. The shape of an SO_4_^2−^ ligand is very close to a regular tetrahedron with a Sulfur (S) atom in the middle and four Oxygen (O) atoms on the vertices, respectively. These four Oxygen atoms are symmetrical so that there are 24 (= 4!) possible ways to match two SO_4_^2−^ ligands in total. Therefore, for the SO_4_^2−^ ligand, we use permutation-distance as the metric to measure the distance between two ligands. We compute the root-mean-square deviations (RMSDs) of all the 24 possible matches, and then choose the smallest RMSD as the permutation-distance between two ligands. Another distance metric is the center-distance, which is the Euclidean distance between the S atom of the current ligand and the true ligand position. We will use both distances to evaluate the actor model performance for multi-atom ligand condition.

The actor model structure in the SO_4_^2−^-ligand dataset experiment is different from the structure in the Cu-ligand dataset experiment. Figure 4 shows the actor model architecture. The filter sizes in the parallel convolutional layer are changed from (2, 2, 2), (3, 3, 3), (4, 4, 4) to (4, 4, 4), (5, 5, 5), (6, 6, 6). Two reasons for this change: First, the SO_4_^2−^ ligand is composed of 5 atoms, which is much larger than the Cu ligand. Larger filters can better capture the features of the SO_4_^2−^ ligand. Second, there is a large space between the SO_4_^2−^ ligand and protein structure in many protein–ligand complexes so that larger filters can detect this property and improve the model performance. It is necessary to consider the rotation of the ligand in the SO_4_^2−^-ligand dataset experiment. In addition to the six possible directions for the translation, we need to output extra six possible directions for the rotation: rotate clockwise and counterclockwise on each of X, Y, and Z axes. The sequential structure will be divided into two parallel branches at the end of the structure, and two 6-dimensional vectors will be output to predict the translation and rotation simultaneously. The critic model also has six layers, with the same first four layers as the actor model. In contrast, there is only one fully connected layer as the fifth layer with unit size 256 and activation ReLU. It is followed by the output layer with 1 unit and activation “tanh”.

## Results

In this section, we will provide the training and test results of the Cu-ligand dataset and SO_4_^2−^-ligand dataset.

### Predicting copper binding

**Fig. 5:**
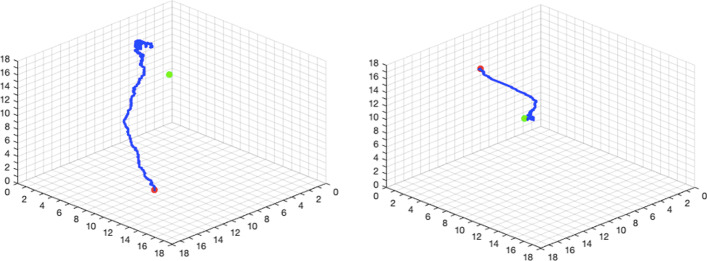
Two searching paths in $18\times 18\times 18$ cubic boxes during the training process for the Cu-ligand dataset. Left: The searching path in episode 10,000. Right: The searching path in episode 20,000. The red point is the start ligand site, the green point is the true ligand site, and the blue path is the searching path. Environment atoms are not presented in plots

**Fig. 6:**
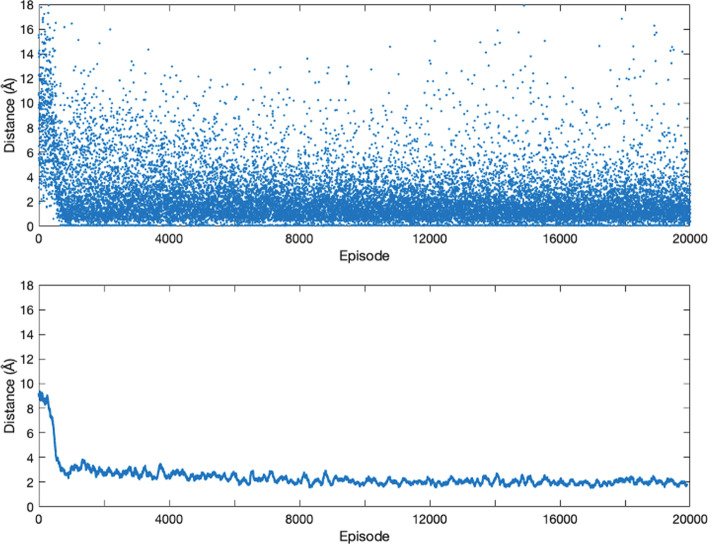
The plots of the last position distances. Upper: The scatter plot of the last position distances. Lower: The smoothed curve of the Upper plot. The X-axis denotes the episode and the Y-axis denotes the distance value

We created 20,000 training samples for ${\mathrm{Cu}}^{2+}$ ligands by placing ${\mathrm{Cu}}^{2+}$ away from their binding sites. As described in the Method section, the actor model will learn the optimal moving direction during the training process, which will lead the ligand towards the true binding site from a distant starting location. Figure 5 shows two examples of the search paths during the training process. The last locations of the ligand are much closer to the binding site (green dots) than the starting locations (red dots). We use the root-mean-square-deviation (RMSD) between the final position of the ligand in the search path and the true position in the PDB file as the measure of model performance where the position of a ligand is defined by the coordinates of all its atoms. A zero RMSD indicates a perfect prediction. Figure 6 shows the RMSDs for 20,000 training samples where the x-axis is the index of the samples in the training process (sample 1 was trained first, and sample 20,000 was trained the last). The RMSD decreases sharply in the first 1,000 episodes and then slowly converges to about 2 Å, indicating that the actor model is able to gradually learn better move strategies with an increasing amount of training data. On average RMSD decreases from around 9 to 2 Å, which is a significant improvement. We compute the “improvement rate”, defined as follows:

$$\frac{{{\text{RMSD}}\left( {s_{0} ,s_{start} } \right) - {\text{RMSD}}\left( {s_{0} ,s_{end} } \right)}}{{{\text{RMSD}}\left( {s_{0} ,s_{start} } \right)}}$$

where ${s}_{0}$ is the true ligand position, ${s}_{\mathrm{start}}$ is the starting position of the ligand on the search path, ${s}_{\mathrm{end}}$ is the last position of the ligand on the search path, $RMS\mathrm{D}(\bullet ,\bullet )$ is the RMSD between two ligand positions, and the position of a ligand is defined by the coordinates of all its atoms. The improvement rate measures the percentage of reduction in terms of the RMSD between the starting position and the true position of the ligand. Table 4 summarizes the means and medians of RMSDs from episode 10,000 to episode 20,000 and the improvement rate in the training and test dataset. In this study, the critic model is used to estimate the true long-term reward. Given the search path, the long-term reward, $G_{t}$, of state $s_{t}$ is:

$$G_{t} = \exp \left\{ { - \frac{{{\text{RMSD}}\left( {s_{0} ,s_{end} } \right)}}{18}} \right\} - \exp \left\{ { - \frac{{{\text{RMSD}}\left( {s_{0} ,s_{t} } \right)}}{18}} \right\}.$$

**Table 4:** The means and medians of the RMSD and improvement rate in the training process and test process for Cu-ligand dataset

	Training dataset		Test dataset	
	Mean	Median	Mean	Median
RMSD	2.0566	1.5183	3.1816	2.5215
Improvement rate	76.46%	83.87%	62.13%	73.15%

If we use the critic model prediction $O_{t}$ to replace $G_{t}$, the $RMS{\text{D}}\left( {s_{0} ,s_{t} } \right)$ can be represented as:

$$- 18 \times \log \left( {\exp \left\{ { - \frac{{{\text{RMSD}}\left( {s_{{{\text{end}}}} ,s_{0} } \right)}}{18}} \right\} - O_{t} } \right).$$

**Fig. 7:**
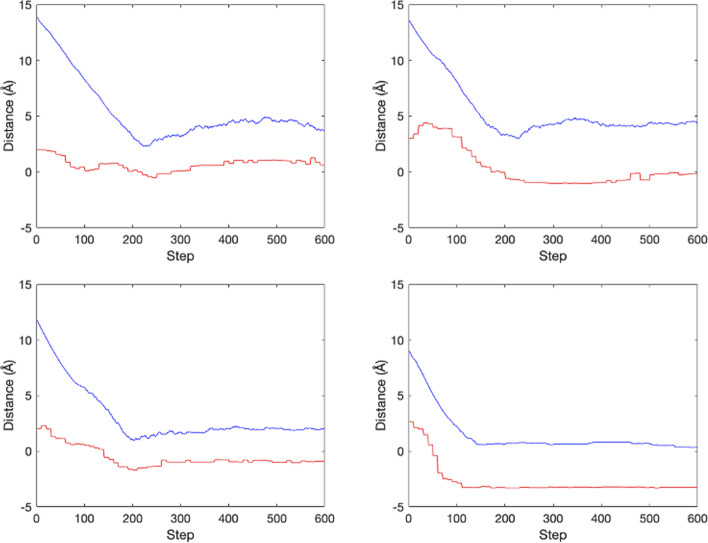
Four cases of the critic distances and the real distance in the training process for Cu-ligand dataset. Upper left: Episode 10,000. Upper right: Episode 13,000. Lower left: Episode 16,000. Lower right: Episode 20,000. The red curve is the critic model predicted distance between the current state and the true ligand site. The blue curve is the real distance between the current state and the true ligand site. The X-axis denotes the step number in the episode and the Y-axis denotes the distance value

This formula describes the distance between the current state and the true ligand position predicted by the critic model, called critic distance. Four examples of the critic distances and real distances between the current and true sites are shown in Fig. 7. Although there is a gap between the true and predicted distances, they have a very similar trend during the training process. This property is used to decide when to stop searching in the test process: if the critic distance is not improved over a certain period, then the searching should stop. Detailed procedure is given as follows.

**Fig. 8:**
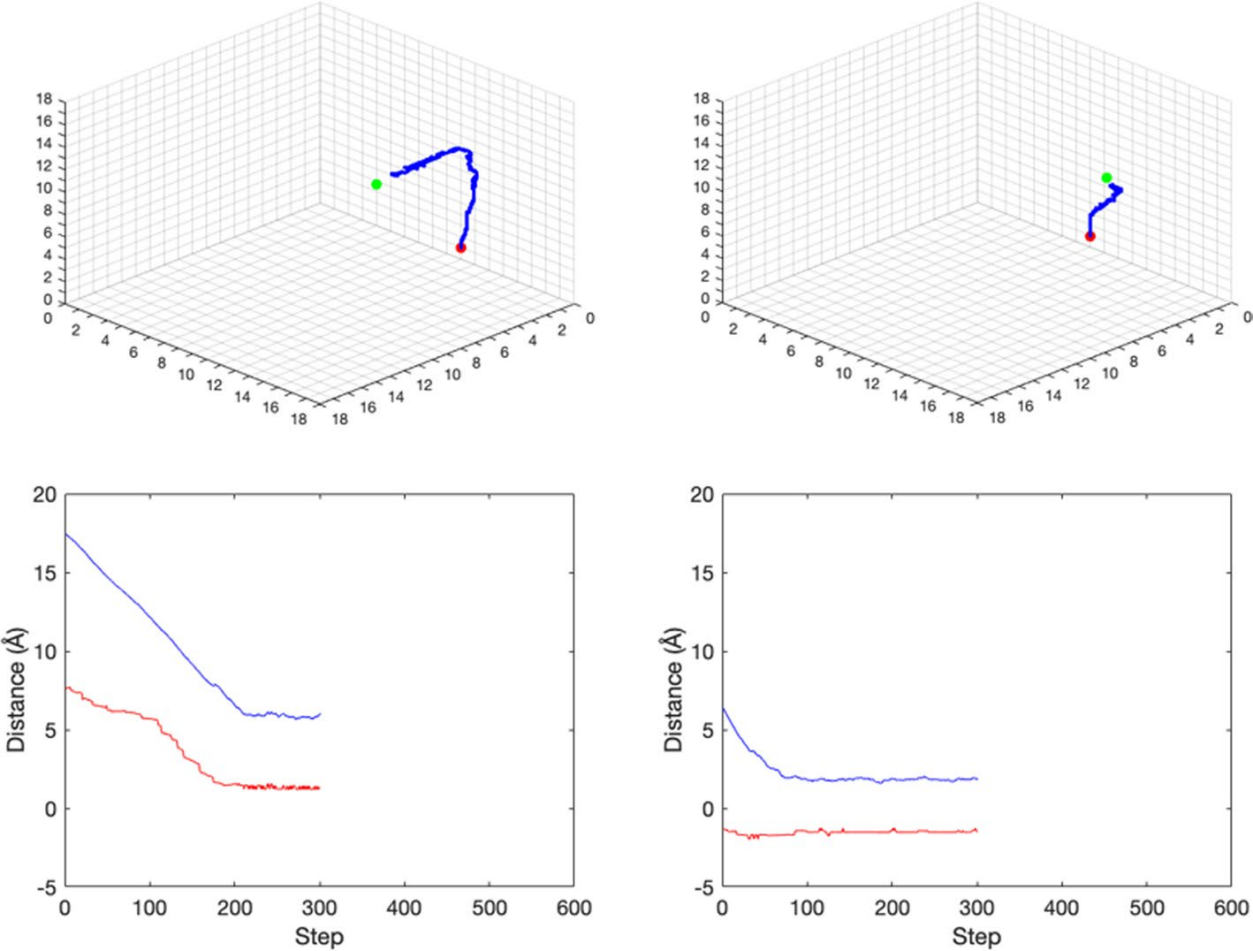
The actor model and critic model performances of Cu-ligand dataset in the test process. Upper left: The searching path in episode 2000. Upper right: The searching path in episode 4000. The red point is the start ligand site, the green point is the true ligand site, and the blue path is the searching path. Environment atoms are not presented in plots. Lower left: The critic distances and the real distance in episode 2000. Lower right: The critic distances and the real distance in episode 4000. The red curve is the critic model predicted distance between the current state and the true ligand site. The blue curve is the real distance between the current state and the true ligand site. The X-axis denotes the step number in the episode. The Y-axis denotes the distance value. Because of the stop criterion, different episodes may have different step numbers

The test process follows Algorithm 2 using 4000 samples. Figure 8 shows the search paths for two samples. Although the searching path in the test process does not reach the true site exactly, the distance between the predicted ligand position and the true ligand position becomes much smaller. In most studies, the protein–ligand interaction problem is depicted as a classification in which we identify corresponding binding locations on an amino acid sequence. Compared with others, our measurement of accuracy focuses on the L_2_ distance between the true and predicted location. The last two columns in Table 4 represent the means and medians of the last position distance and improvement rate for the test process, and both metrics indicate a significant improvement in prediction accuracy compared with a randomly selected position. Our results are comparable with that proposed by DeepPocket [28], which locates potential pockets and utilizes the distance between the predicted and actual center of the pocket, or DCC, to evaluate model performance. Predictions with DCCs less than 4 Å are considered successful. In contrast to an average DCC success rate of 85.2% shown in DeepPocket, our RL model can achieve a result of 81%. What’s more, two examples are shown in Fig. 8 to illustrate the critic model performance in the test process. Based on the plots, a gap still exists between the true and estimated distance curves. However, it is reliable to use the critic model to determine when the ligand should stop. Another advantage of using the critic model as a stopping criterion is its efficiency. In most cases, the episode can stop much earlier than the maximum of 600 steps.

### Sulfate ligand experimental results

Table 5 summarizes the training and test results of the last position permutation-distance and the last position center-distance and improvement rates for both two distances in the sulfate-ligand dataset. The training process involves episode 10,000 to episode 24,900, and the test process uses all 4600 episodes. The center-distance results are all better than the permutation-distance results, but in general, the SO_4_^2−^-ligand dataset experimental results are not as good as that for Cu-ligand. We can see the average improvement rate for center-distance in SO_4_^2−^-ligand test data is 48.08%. On the contrary, the average improvement rate of Cu-ligand test case is 62.13%. One possible reason is the structural complexity of Sulfate ligand. Protein–ligand complexes with the SO_4_^2−^ ligand are more complex, and the models are more difficult to capture sufficient information. Another reason is that a large space is found around the Sulfate ligand true ligand position, whereas the actor model prefers to sites around atoms. However, we emphasize that the improvement rate in the SO_4_^2−^ ligand dataset still indicates the success of the proposed framework. The effect of the rotation is hard to observe since there is still a large gap between the predicted ligand position and the true ligand position. In addition, it is still applicable to use the critic distance as a stopping criterion because it shows the same tendency as the real distance curve.

**Table 5:** The means and medians of the last position distance and improvement rate in the training process and test process for the SO_4_^2−^-ligand dataset

	Training dataset		Test dataset	
	Mean	Median	Mean	Median
The last position permutation-distance	4.2566	3.8366	4.4347	3.9361
The last position center-distance	4.1057	3.7129	4.2810	3.8191
Improvement rate for permutation-distance	49.00%	59.22%	48.08%	58.80%
Improvement rate for center-distance	50.08%	60.85%	49.12%	60.01%

### Further analysis on binding specificity

Considering the fact that both copper and sulfate ions can be found at nonspecific sites on a protein surface with no known biological function, we split out test samples into specific and nonspecific binding group by comparing the shortest distance between the ion and specific atoms in the protein and vdW bond [29]. This split is straightforward in the ${\mathrm{Cu}}^{2+}$ data, while significantly more challenging in the SO_4_^2−^ data. Hence, we restrict our samples to the copper-ligand. We then perform the same prediction steps as described for the overall data, and the result in the test data is given in Table 6 below.

**Table 6:** The model performance on copper-ligand test dataset by binding specificity

	Test dataset			
	Specific binding		Nonspecific binding	
	Mean	Median	Mean	Median
RMSD	2.8875	2.4725	3.9487	2.6334
Improvement rate	66.14%	73.42%	54.11%	70.15%

In the ${\mathrm{Cu}}^{2+}$ data, 75% of test samples are specific binding and the other 25% are set to be nonspecific binding samples. Compared with nonspecific binding samples, the predicted final position for specific binding case is closer to the true location on average, and the improvement rates in both mean and median values are higher. This indicates that our model might be more capable of adopting specific binding patterns and find the corresponding binding location.

## Summary and discussion

In this study, we have proposed a novel framework based on the A3C model for protein–ligand docking (PLD) and tested its feasibility. Using single-atom ligands and a small multi-atom ligand, we showed that the algorithm could move the ligands to locations very close to their true binding sites when starting from random starting locations far away from the proteins. In particular, we point out that our promising results on SO_4_^2−^ demonstrated the potential ability of generalization on more complicated ligands in the future study. In addition, when PDB structures show protein–ligand binding, it can be difficult to distinguish whether it is real binding or artifacts when the binding sites are not functional sites of the protein. In our study, we did not make that distinction. As a result, the performance was probably negative affected. If the artifacts cases can be identified and removed from the dataset, the performance may be improved.

The models built in this study are ligand-specific because we want to start from a simple scenario to see whether the RL would work in this simple case. Another reason for developing ligand-specific models first is because the general model would require a large number of ligands and ligand–protein complexes. Developing such a model would be well beyond the goal of this study, which is to demonstrate the feasibility of applying RL to PLD problem. In fact, ligand-specific models can be quite useful for solvent mapping [24], which has been used widely in structure-based drug design to provide useful information on potential binding pockets on protein surfaces and the corresponding ligand properties. These ligand-specific models can be directly trained as long as there are enough data in PDB for the ligand of interest. It would be an interesting topic for future studies to investigate the amount of data necessary for building a ligand-specific model, which is likely dependent also on the type of ligands.

In this study, we used a carefully designed Convolutional Neural Network (CNN) architecture to learn both the actor and critic models. More sophisticated architectures can also be used such as DenseNet, ResNet, and various attention mechanisms [30], Kimber et al. [31], Kandel et al. [32]. We expect the performance to be further improved with the latest deep learning architectures.

One of the future studies would be to combine all the ligands together and train a general model for protein ligand docking. Such model would require the model to learn more specific interactions among different atoms on both proteins and the ligands. To extend out framework to more general applications, we first need to add more atom types for ligands (Sciortino et al. [33, 34], and increase the dimension of the input data to accommodate more atoms on the ligands. In addition, we will need to train such a model using training data from more databases such as Binding MOAD [35] and CCDC/Astex [36].

We emphasize that the proposed RL framework is still preliminary. Apart from what has been covered, alternative methods, more datasets and more types of proteins, and detailed comparison with the state-of-the-art would be explored in the future. In particular, we could collect multiple history steps in a set, called the replay buffer [37], and draw a batch of de-correlated samples to perform mini-batch gradient descent. Such an approach could accelerate the learning speed and training efficiency.

## Data Availability

The datasets generated and/or analysed during the current study are available in the RSCB The Protein Data Bank https://www.rcsb.org/
